# Hematological Profile in Sickle Cell Disease: A Systematic Review and Meta‐Analysis Comparing Steady‐State and Vaso‐Occlusive Crisis Phases (2000–2025)

**DOI:** 10.1155/bmri/2741454

**Published:** 2026-04-17

**Authors:** Romaric De Manfouo Tuono, Josué Louokdom Simo, Ingrid Worti Sulem Yong, Maryline Seuko Njopwouo, Claude Tagny Tayou, Paul Koki

**Affiliations:** ^1^ Higher Institute of Health Sciences, University of Montagnes, Bangangté, Cameroon; ^2^ Faculty of Medicine and Biomedical Sciences, Université de Yaoundé 1, Yaoundé, Cameroon, uninet.cm; ^3^ Centre Mère Enfant de la Fondation Chantal Biya, Yaoundé, Cameroon

**Keywords:** complete blood count profile, sickle cell disease, steady state, vaso-occlusive crisis

## Abstract

**Background and Aims:**

Sickle cell disease (SCD) is a genetic disorder characterized by intrinsic clinical manifestations, frequently exacerbated by vaso‐occlusive episodes (VOEs), which significantly affect hematological parameters. This systematic review and meta‐analysis is aimed at synthesizing evidence from studies published over the past 25 years on complete blood count profiles in SCD patients, both in the steady state and during VOEs. The objective was to clarify hypotheses and highlight the need for prospective studies to establish specific reference values for steady‐state SCD patients, thereby improving patient management.

**Materials and Methods:**

In accordance with the PRISMA guidelines and the Cochrane Handbook, a systematic search of major databases (including PubMed, Web of Science, and Google Scholar) was conducted to identify analytical studies published between 2000 and 2025. Eligible studies compared complete blood count profiles in SCD patients during VOEs and in the steady state. Study selection and data extraction were performed independently by two reviewers, with disagreements resolved by consensus. Extracted data were entered into Microsoft Excel 2013 and analyzed using R statistical software (Version 4.3.2). Between‐study heterogeneity was evaluated using Higgins′ inconsistency (*Q*) statistics, with results expressed as *I* ^2^ values and corresponding *p* values. The risk of bias in the included studies was assessed using the ROBINS‐E tool.

**Results:**

Eight studies met the inclusion criteria. Pooled analyses demonstrated a significant impact of VOEs on hemogram parameters. This impact was characterized by anemia and erythrocytopenia (standardized mean difference [SMD], random‐effects model: −0.97 [−1.45; −0.50]), accompanied by microcytosis (SMD, random‐effects model: 0.18 [−0.21; 0.58]) and hypochromia (SMD, random‐effects model: −0.90 [−2.96; 1.15]). These changes were also associated with reduced fetal hemoglobin levels, increased red cell distribution width (RDW), and reticulocytosis. Regarding the white blood cell lineage, VOEs were associated with leukocytosis (SMD, random‐effects model: 0.73 [0.23; 1.24]) and a moderate increase across leukocyte subpopulations. Concerning the platelet lineage, VOEs exerted variable effects on platelet production, ranging from negative to positive influences, with an overall increase in platelet count (SMDs, random‐effects model: −0.17 [−1.13; 0.78] and 0.78 [0.53; 1.03], respectively), accompanied by reductions in mean platelet volume and platelet distribution width (PDW).

**Conclusion:**

This meta‐analysis confirms the substantial influence of VOEs on red blood cell, white blood cell, and platelet parameters, as well as their associated indices. Importantly, although hematological parameters in the steady state are less severely altered than during VOEs, they often remain outside conventional reference ranges, reflecting a distinct physiological baseline in SCD patients. These findings underscore the urgent need for prospective studies to define specific hematological reference values for steady‐state SCD patients, which is essential for optimizing their clinical management.

## 1. Introduction

Sickle cell disease (SCD) is an inherited hematological disorder characterized by the production of abnormal hemoglobin S, which deforms red blood cells (RBCs) into a sickle shape [1, 2]. This morphological alteration increases RBC rigidity and impairs their passage through microvessels, leading to vaso‐occlusive episodes (VOEs), tissue damage, and chronic inflammation [3]. Globally, approximately 312,000 homozygous SCD births occur annually, with the vast majority (236,000) concentrated in sub‐Saharan Africa [4, 5]. SCD thus represents a major global public health challenge, requiring coordinated and urgent management strategies [6].

Effective patient care demands a multifactorial and multidisciplinary approach that considers the various systems affected by the multiple complications to which SCD patients are predisposed [7, 8]. Regardless of the system involved, the primary biological monitoring tool that provides an overview of the patient′s general condition is the complete blood count (CBC) [9, 10]. The CBC allows for the evaluation of the quantity and quality of the three main blood lineages: RBCs (erythrocytes), white blood cells (leukocytes), and platelets [11, 12]. This test is essential for diagnosing anemia, hematological malignancies, infections, acute hemorrhagic states, allergies, and immunodeficiencies [13].

Several factors considerably influence CBC results in SCD patients, with hemolytic and vaso‐occlusive crises being particularly significant [1, 14]. Although infections, vaso‐occlusive events, and anemic crises are common in children with SCD, vaso‐occlusion has been reported as the most frequent manifestation and the leading cause of hospitalization, organ failure, and mortality in this population [15]. Vaso‐occlusion, which drives multiple pathogenic mechanisms responsible for numerous complications, is primarily caused by the adhesion of RBCs to the vascular endothelium, coupled with anemia, leukocyte activation, and platelet aggregation, resulting in elevated values that may predispose to complications [5].

The steady‐state period is defined as “a crisis‐free interval extending from at least three weeks after the last clinical event and 3 months or more after the last blood transfusion, up to at least 1 week before the onset of a new clinical event” [16]. Accurate interpretation of the hemogram in SCD patients remains challenging and requires reference values tailored not only to SCD patients in general but, more importantly, to those in the steady state [13]. Currently, interpretations often rely on standard WHO reference ranges [17]; however, steady‐state patients frequently display hematological values considered “abnormal” by these conventional standards, despite being clinically stable [11]. For example, Diallo et al. [18] reported a significantly lower mean platelet count during acute attacks (401.13 ± 138 G/L) compared with the intercritical phase (456.15 ± 164 G/L; *p* = 0.02), and a significantly higher prevalence of thrombocytosis during the intercritical phase (*p* = 0.001) [18].

These observations highlight the need to establish a specific hematological profile for SCD patients in the steady state. In resource‐limited settings, where patient care and follow‐up are particularly challenging, defining such reference values is crucial for optimizing management and anticipating potential complications. This study is aimed at reviewing hemogram profiles reported in SCD patients over the past 25 years, both during VOEs and in stable conditions, in order to propose hypotheses and perspectives for prospective studies to define steady state–specific reference values. This approach could ultimately improve the management of SCD patients in Cameroon, across Africa, and globally.

The clinical utility of this meta‐analysis lies in identifying objective biological signatures of VOEs. Consistent changes in CBC parameters between crisis and steady state may serve as a biological validation of acute episodes and support clinical decision‐making.

## 2. Methods

This systematic review and meta‐analysis was conducted to assess the hemogram profile in sickle cell patients during both critical (VOEs) and steady‐state phases. Its primary objective is to inform the hypothesis and rationale for conducting prospective studies to establish specific hematological reference values for steady‐state SCD patients, aimed at enhancing their clinical management.

### 2.1. Search Strategy and Study Selection

Articles were retrieved from the Google Scholar, Medline–PubMed, and Web of Science databases, selected for their rigorous indexing. The search strategy employed Medical Subject Headings (MeSH) terms and combinations of keywords, including: “sickle cell disease AND blood count profile,” “blood count profile AND sickle cell disease in steady state,” “blood count profile AND sickle cell disease in steady state AND vaso‐occlusive crises,” and “sickle cell disease AND primary hemostasis.” Synonyms such as CBC and hemogram were also used. The search was limited to studies published between 2000 and 2025. Only studies that met the predefined objectives of this review were included.

### 2.2. Selection Criteria for Studies and Data Extraction

Inclusion criteria encompassed analytical studies comparing haemogram profiles in SCD patients during both steady‐state and critical (VOEs) phases. Patients in the included studies were exclusively HbSS. Studies involving patients with different genotypes were not included. Two independent, qualified reviewers initially screened the titles and abstracts of all retrieved publications. Full texts of potentially relevant studies were then independently assessed against the inclusion criteria. Only studies indexed in English were considered. Studies meeting the initial screening were subjected to a peer‐review process before final inclusion. Any disagreements between reviewers were resolved by consensus.

Data extraction was performed using a preformatted Microsoft Excel spreadsheet, with each variable clearly labeled at the top of each column to ensure structured and coordinated extraction. Extracted data were subsequently verified for accuracy and consistency. Information collected from each article included article identification details (DOI and journal name), study characteristics (authors, country, continent, year of publication, and study type), population characteristics (sample size, age, and sex), study methods (design, tools, and interventions), and main outcomes.

### 2.3. Outcome Measurement

The CBC allows for the assessment of both the quantity and quality of the three blood lineages: erythrocytes (RBCs), leukocytes (white blood cells), and platelets [11, 12]. Major factors such as vaso‐occlusive and anemic crises significantly influence this profile, and the final outcomes may vary depending on the presence or absence of a crisis [1, 14].

The parameters used to define these outcomes in SCD patients were as follows:

• Steady‐state period: Defined as the period free of crisis, extending from at least 3 weeks after the last clinical event and 3 months or more since the last blood transfusion, up to at least 1 week before the onset of a new clinical event [16].
• RBC line and erythrocyte parameters: RBCs (4–6 × 10^6^/*μ*L), hemoglobin (12–17 g/dL), hematocrit (HCT) (35%–55%), reticulocytes (50–120 G/*μ*L), mean corpuscular volume (MCV) (80–100 fL), mean corpuscular hemoglobin (MCH) (27–29 pg), mean corpuscular hemoglobin concentration (MCHC) (32–36 g/L), red cell distribution width (RDW) (11%–14%) [19, 20];
• White blood cell line: Total leukocytes (4000–10,000/*μ*L), lymphocytes (1000–4000/*μ*L), monocytes (200–1000/*μ*L), neutrophils (2000–7000/*μ*L), eosinophils (50–500/*μ*L), and basophils (0–100/*μ*L) [19].
• Platelet line and parameters: Platelets (150–400 G/*μ*L), mean platelet volume (MPV) (7–11 fL), platelet distribution width (PDW) (10%–17%), plateletcrit (PCT) (0.15%–0.35%) [20].

### 2.4. Risk of Bias Assessment

**Figure 1:**
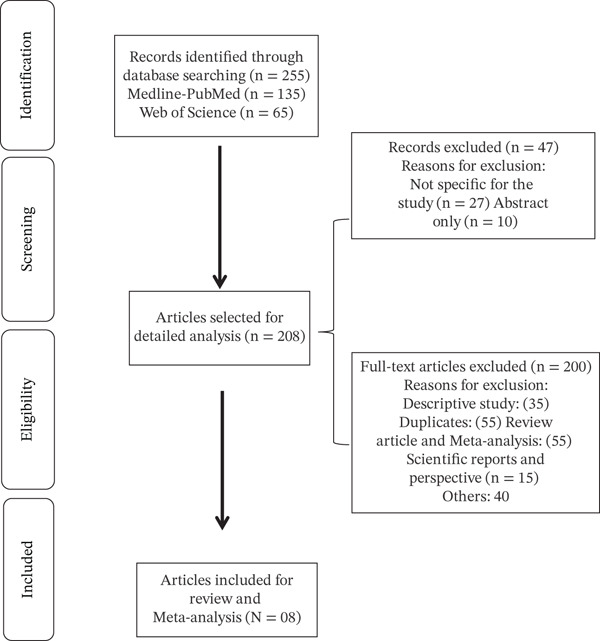
Flow diagram of the literature search.

The risk of bias in the included studies was evaluated using ROBINS‐E, a tool designed to assess bias in nonrandomized studies of exposure effects [21]. ROBINS‐E examines seven domains of bias, as illustrated in Figure 1. Each domain is assessed through a set of signaling questions intended to elicit relevant information about the study design and analytical approach. Most questions allow the response options “yes,” “probably yes,” “probably no,” “no,” and “no information.” Responses of “yes” and “probably yes” carry the same implications for risk of bias, as do “no” and “probably no” [21].

The domains evaluated included bias due to confounding, bias related to exposure measurement, bias in the selection of participants into the study or analysis, bias arising from postexposure interventions, bias due to missing data, bias related to outcome measurement, and bias in the selection of the reported result. All data were entered into a Microsoft Excel 2016 spreadsheet and analyzed using the aforementioned tool.

### 2.5. Data Synthesis, Management, and Analysis

The statistical analyses were performed using R software, Version 4.3.2 (The R Foundation for Statistical Computing, Vienna, Austria). All the included studies compared data between patients in VOEs and those in the steady‐state phase. Extracted data from the included articles were analyzed and presented as forest plots, comparing results from patients during VOEs with those in the steady‐state phase. Each outcome was accompanied by a narrative description.

The mean difference and effect size between the experimental group (patients in VOEs) and the control group (patients in the steady‐state phase) were quantified using the standardized mean difference (SMD), as defined by Wan et al. (2014) in their study *“*Estimating the Sample Mean and Standard Deviation From the Sample Size, Median, Range, and/or Interquartile Range*”* [22]. Interpretation of SMD values was as follows: 0.2 ≤ SMD < 0.3: small effect, 0.3 ≤ SMD < 0.8: moderate effect, and SMD ≥ 0.8: large effect. Positive SMD values indicate an increase in the evaluated parameter in the experimental group, whereas negative values indicate a decrease [23]. Heterogeneity among individual studies was assessed using Higgins′ *Q* statistic, reported as *I* ^2^ with an associated *p* value [24]. Heterogeneity was classified as low: *I* ^2^ < 25*%*, moderate: *I* ^2^ = 25*%*–50*%*, and high: *I* ^2^ > 50*%* [24]. All analyses were performed with a 95% confidence interval, and *p* values < 0.05 were considered statistically significant.

## 3. Results

### 3.1. Revision Process

**Figure 2:**
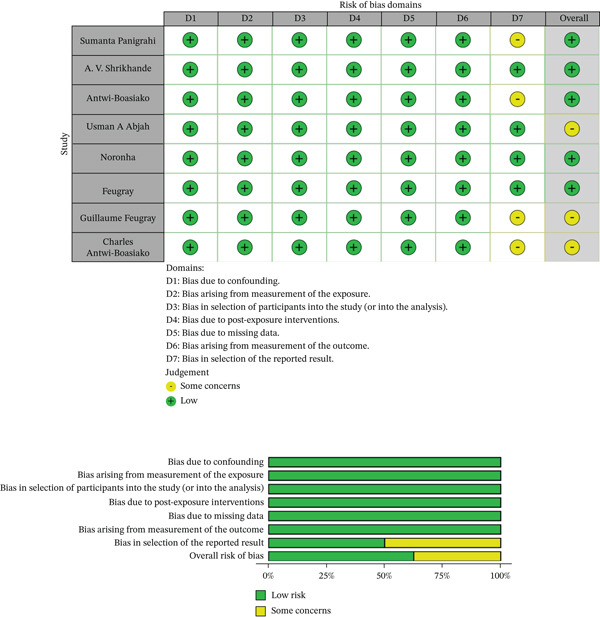
Risk of bias assessment of nonrandomized studies of exposure effects using ROBINS‐E [21].

The initial search using specific keywords yielded 255 articles across the selected databases: 55 from Google Scholar, 135 from Medline–PubMed, and 65 from Web of Science. From this preliminary set, 47 articles were excluded because the full text was unavailable or the study objectives were not aligned with our review, leaving 208 articles for further assessment of study type and design. Subsequently, duplicates, review articles, perspective pieces, scientific reports, meta‐analyses, and descriptive studies were identified and removed. Ultimately, eight studies fulfilling the primary objectives of this review were retained for inclusion (Figure 2).

### 3.2. Results of Risk of Bias Assessment

Figure 2 presents the results of the risk of bias assessment conducted using the ROBINS‐E tool.

Following the assessment, although some studies showed “some concerns,” the overall risk of bias across all included studies was generally rated as “low” (as illustrated in Figure 2). Heterogeneity was moderate, with an *I* ^2^ of approximately 60%, thereby supporting and justifying the presence of statistically significant differences between individuals with SCD (HbSS) and control subjects (HbAA).

### 3.3. Characteristics of the Included Studies

The search period for the included studies spanned from 2000 to 2025. All selected studies were analytical case‐control studies, with the case group comprising sickle cell patients in the episodes (VOEs) phase and the control group comprising sickle cell patients in the steady‐state phase. The study populations included individuals of all ages and both sexes. In total, eight studies met the inclusion criteria, with the distribution of the included studies as follows:

- Three from Africa with the following authors and years of publication: Antwi‐boasiako et al. [10] from Ghana, Abjah et al. [25] from Nigeria, and Antwi‐Boasiako et al. [26] from Ghana;
- Two from Asia and India by authors Panigrahi et al. [27] and Shrikhande et al. [12];
- One study of America, Brazil, and the authors are Noronha et al. [28]; and
- Three studies of Europe, all from France, by authors Feugray et al. [29] and Feugray et al. [30].

### 3.4. Results of the Included Studies

#### 3.4.1. Red Lineage Profile

##### 3.4.1.1. RBCs and Erythrocyte Indices.

###### 3.4.1.1.1. RBCs.

**Figure 3:**
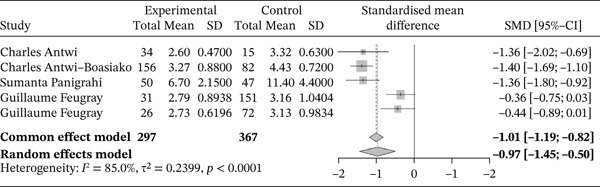
Forest plot showing red blood cells.

The figure below presents a forest plot illustrating the comparison of RBC counts between sickle cell patients during VOEs and those in the steady‐state phase (Figure 3).

Of these, five studies compared RBC counts in sickle cell patients during VOEs versus the steady‐state phase, encompassing a total of 664 participants, 297 in crisis and 367 in the steady state. The SMD, calculated using a random‐effects model, was −0.97 (−1.45; −0.50), indicating a substantial reduction in RBC counts during VOEs. The study showing the largest effect was Feugray et al. [30] (−0.36 [−0.75; 0.03]). Heterogeneity among the included studies was high (*I* ^2^ = 85*%*), suggesting considerable variability in the observed effects.

###### 3.4.1.1.2. MCV.

**Figure 4:**
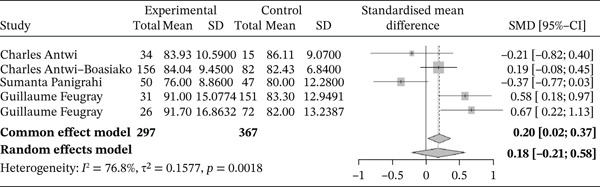
Forest plot showing the MCV.

Figure 4 shows the forest plot representation of studies comparing MCV in sickle cell patients during VOEs and those in the stable phase.

Of these, five studies compared the MCV in sickle cell patients during VOEs and in the steady phase, including a total of 664 participants (297 patients in VOEs and 367 in steady state). The SMD calculated using the random‐effects model was 0.18 (−0.21; 0.58), suggesting a minimal, nonsignificant effect of VOEs on MCV compared with controls. The study reporting the largest effect was that of Antwi‐Boasiako et al. [26] (−0.21 [−0.82; 0.40]). Heterogeneity among the included studies was high, with *I* ^2^ = 76.8*%*.

###### 3.4.1.1.3. MCHC.

**Figure 5:**
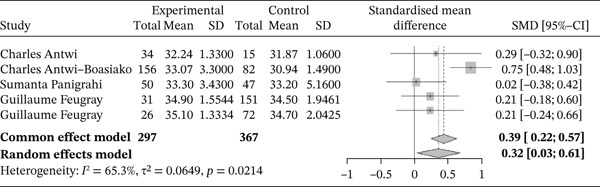
Forest plot presenting the MCHC.

Figure 5 shows the forest plot representation of studies comparing the mean MCHC in sickle cell patients during VOEs and in the steady phase.

Of these, five studies compared the mean MCHC in sickle cell patients during VOEs and in the steady phase, involving a total of 664 participants, including 297 patients in VOEs and 367 in the steady phase. The SMD calculated using the random‐effects model was 0.32 (0.03; 0.61), indicating a low to moderate impact of VOEs on MCHC compared with controls. The study reporting the greatest effect was that of Antwi‐Boasiako et al. [10] (0.75 [0.48; 1.03]). Heterogeneity among the included studies was moderate, with *I* ^2^ = 65.3*%*.

###### 3.4.1.1.4. MCH.

**Figure 6:**
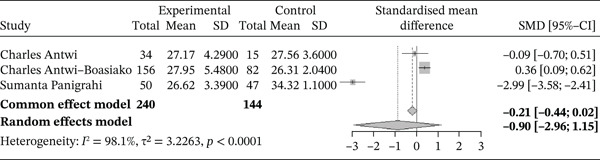
Forest plot showing the MCH.

Figure 6 below shows the forest plot representation of studies comparing MCH in sickle cell patients during VOEs and in the steady phase.

Of these, three studies compared MCH in sickle cell patients during VOEs and in the steady phase, including a total of 384 participants: 240 patients in VOEs and 144 in the steady phase. The SMD calculated using the random‐effects model was −0.90 (−2.96; 1.15), reflecting a significant negative influence of VOEs on MCH. The study reporting the greatest effect was that of Panigrahi et al. [27] (−2.99 [−3.58; −2.41]). Heterogeneity among the included studies was very high, with *I* ^2^ = 98.1*%*.

###### 3.4.1.1.5. HCT.

**Figure 7:**
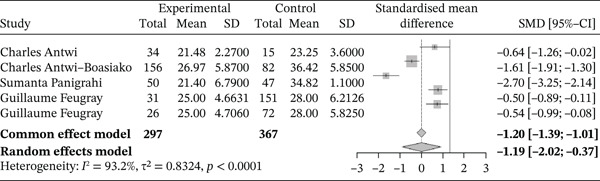
Forest plot showing hematocrit.

Figure 7 below shows the forest plot representing studies that compared HCT in sickle cell patients during events and those in the stable phase.

Of these, five studies compared HCT in sickle cell patients during VOEs and in the steady phase, including a total of 664 participants: 297 patients in VOEs and 367 in the steady phase. The SMD calculated using the random‐effects model was −1.19 (−2.02; −0.37), indicating a significantly negative impact of VOEs on HCT compared with controls. The study reporting the largest effect was that of Panigrahi et al. [27] (−2.70 [−3.25; −2.14]). Heterogeneity among the included studies was high, with *I* ^2^ = 93.2*%*.

###### 3.4.1.1.6. RDW.

**Figure 8:**
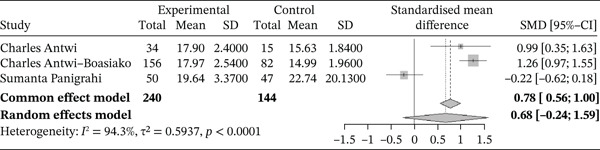
Forest plot showing the RDW.

Figure 8 below shows the forest plot representing studies that compared RDW in sickle cell patients during VOEs and those in the stable phase.

Of these, three studies compared RDW in sickle cell patients during VOEs and those in the steady phase, involving a total of 388 participants, including 240 patients in VOEs and 148 patients in the steady phase. The SMD reported using the random‐effects model is 0.68 (−0.24; 1.59), reflecting a significantly positive effect of VOEs on RDW elevation. The study reporting the greatest effect is that of Antwi‐Boasiako et al. (2018) [10] (2.70 [−3.25; −2.14]). Heterogeneity among the included studies is *I* ^2^ = 94.3*%*.

##### 3.4.1.2. Hemoglobin, Fetal Hemoglobin (HbF), and Reticulocytes.

###### 3.4.1.2.1. Hemoglobin.

**Figure 9:**
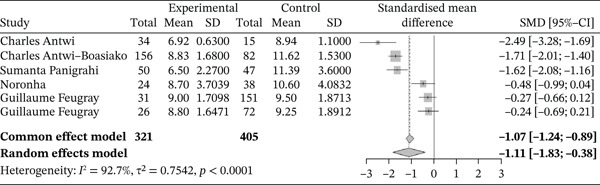
Forest plot showing hemoglobin levels.

Figure 9 shows the forest plot representation of studies comparing hemoglobin levels in sickle cell patients during VOEs and those in the steady phase.

Of these, six studies compared hemoglobin levels in sickle cell patients during VOEs and those in the steady phase, including a total of 726 participants (321 patients in crisis and 405 patients in the steady phase). The SMD reported using the random‐effects model is −1.11 (−1.83; −0.38), indicating a significantly negative impact of VOEs on hemoglobin levels. The study reporting the largest effect was that of Antwi‐Boasiako et al. [10] (−2.49 [−3.28; −1.69]). Heterogeneity among the included studies was high (*I* ^2^ = 92.7*%*).

###### 3.4.1.2.2. HbF.

**Figure 10:**
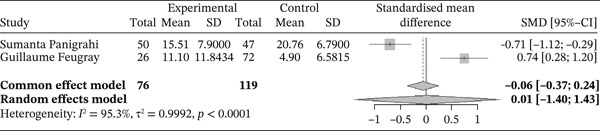
Forest plot showing fetal hemoglobin.

Figure 10 below shows the forest plot representation of studies comparing HbF levels in sickle cell patients during VOEs (cases) and those in the steady phase (controls).

Of these, two studies compared HbF levels in sickle cell patients during VOEs and those in the steady phase, for a total of 195 participants, including 76 patients in crises and 119 patients in the steady phase. The SMD reported using the random‐effects model is 0.01 (−1.40; 1.43), indicating a negligible influence of VOEs on HbF levels. The study reporting the greatest effect is that of Panigrahi et al. [27] (−2.49 [−3.28; −1.69]). Heterogeneity among the included studies is high, with *I* ^2^ = 95.3*%*.

###### 3.4.1.2.3. Reticulocytes.

**Figure 11:**
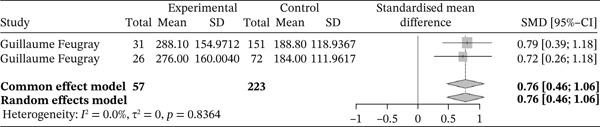
Forest plot showing reticulocytes.

Figure 11 below shows the forest plot representation of studies comparing reticulocyte counts in sickle cell patients during VOEs and those in the steady phase.

Of the latter, two studies compared reticulocyte counts in sickle cell patients during VOEs and those in the steady phase, for a total of 280 participants, including 57 patients in crises and 223 patients in the steady phase. The SMD reported using the random‐effects model is 0.76 [0.46; 1.06], reflecting a significantly positive influence of VOEs on reticulocyte production. The study reporting the greatest effect is that of Feugray et al. [30] (0.79 [0.39; 1.18]). Heterogeneity among the included studies is *I* ^2^ = 0.0*%*.

#### 3.4.2. White Blood Cells and Leukocyte Formula

##### 3.4.2.1. White Blood Cells.

**Figure 12:**
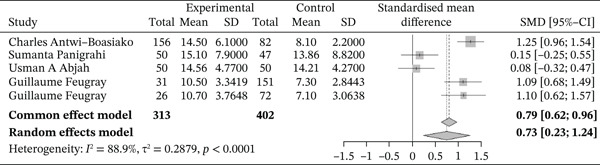
Forest plot showing white blood cells.

Figure 12 below shows the forest plot representation of studies comparing white blood cell counts in sickle cell patients during VOEs and those in the steady phase.

Of these, five studies compared white blood cell counts in sickle cell patients during VOEs and those in the steady phase, for a total of 715 participants, including 313 patients in VOEs and 402 patients in the steady phase. The SMD reported using the random‐effects model was 0.73 [0.23; 1.24], indicating a significantly positive influence of VOEs on white blood cell production. The study reporting the greatest effect was that of Antwi‐Boasiako et al. [10] (1.25 [0.96; 1.54]). Heterogeneity among the included studies was high (*I* ^2^ = 88.9*%*).

##### 3.4.2.2. Lymphocytes.

**Figure 13:**
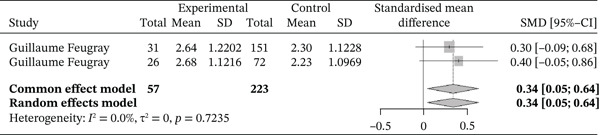
Forest plot showing lymphocytes.

Figure 13 below shows the forest plot representation of studies comparing lymphocyte counts in sickle cell patients during VOEs and those in the steady phase.

Of these, two studies compared lymphocyte counts in sickle cell patients during VOEs and those in the steady phase, for a total of 280 participants, including 57 patients in VOEs and 233 patients in the steady phase. The SMD reported using the random‐effects model was 0.34 [0.05; 0.54], indicating a statistically significant moderate influence of VOEs on lymphocyte production. The study reporting the greatest effect was that of Feugray et al. [30] (0.40 [−0.05; 0.86]). No heterogeneity was observed between the included studies (*I* ^2^ = 0.0*%*).

##### 3.4.2.3. Monocytes.

**Figure 14:**
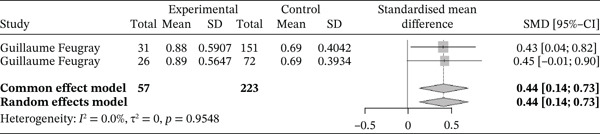
Forest plot showing monocytes.

Figure 14 below shows the forest plot representation of studies comparing monocyte counts in cases and controls.

Of these, two studies compared monocyte counts in sickle cell patients during VOEs and those in the steady phase, for a total of 280 participants, including 57 patients in VOEs and 233 patients in the steady phase. The SMD reported using the random‐effects model was 0.44 (0.14; 0.73), indicating a significantly moderate influence of VOEs on monocyte production. The study reporting the greatest effect was that of Feugray et al. [30] (0.45 [−0.01; 0.90]). Heterogeneity among the included studies was *I* ^2^ = 0.0*%*.

##### 3.4.2.4. Neutrophils.

**Figure 15:**
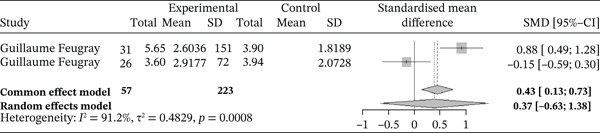
Forest plot présentant les neutrophiles.

Figure 15 below shows the forest plot representation of studies comparing neutrophil counts in sickle cell patients during VOEs and those in the steady phase.

Of these, two studies compared neutrophil counts in sickle cell patients during VOEs and those in the stationary phase, for a total of 280 participants, including 57 patients in VOEs and 233 patients in the stationary phase. The SMD reported using the random‐effects model was 0.37 (−0.63; 1.38), indicating a moderately positive influence of VOEs on neutrophil production. The study reporting the greatest effect was that of Feugray et al. [30] (0.88 [0.49; 1.28]). Heterogeneity among the included studies was high (*I* ^2^ = 91.2*%*).

#### 3.4.3. Platelet Lineage Profile and Platelets Constants

##### 3.4.3.1. Platelets.

**Figure 16:**
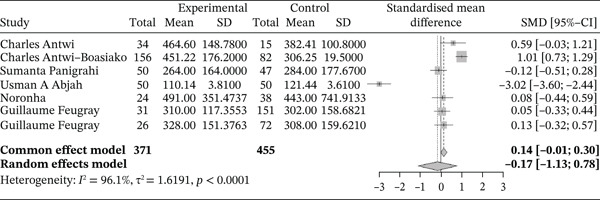
Forest plot showing platelets.

Figure 16 below shows the forest plot representation of studies comparing platelet counts in sickle cell patients during VOEs and those in the stable phase.

Of these, seven studies compared platelet counts in sickle cell patients during VOEs and those in the stationary phase, for a total of 826 participants, including 371 patients in VOEs and 455 patients in the stationary phase. The SMD reported using the random‐effects model was −0.17 (−1.13; 0.78), reflecting a mixed effect ranging from negative to positive influence of VOEs on platelet production. Specifically, Abjah et al. [25] reported a negative influence of VOEs on platelet production (−3.02 [−3.60; −2.44]), whereas Antwi‐Boasiako et al. [10] reported a positive influence (1.01 [0.73; 1.29]). The heterogeneity among the included studies was high (*I* ^2^ = 96.1*%*).

##### 3.4.3.2. MPV.

**Figure 17:**
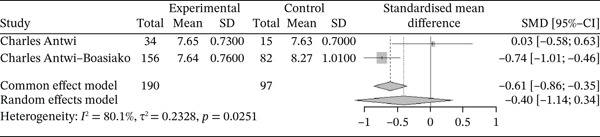
Forest plot showing MPV.

Figure 17 below shows the forest plot representation of studies comparing MPV in sickle cell patients in VOEs and those in stable phase:

Of the latter, two studies compared MPV in sickle cell patients during VOEs and those in the stationary phase, including a total of 287 participants: 190 patients in VOEs and 97 patients in the stationary phase. The SMD reported using the random‐effects model is −0.40 (−1.14; 0.34), reflecting a negative influence of VOEs on MPV. The study reporting the greatest effect is that of Antwi‐Boasiako et al. [10] (−0.74 [−1.01; −0.46]). Heterogeneity between the included studies is *I* ^2^ = 80.1*%*.

##### 3.4.3.3. PDW.

**Figure 18:**
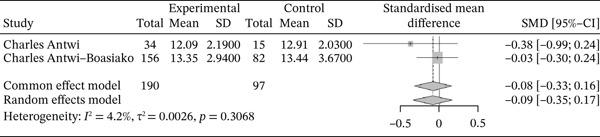
Forest plot showing PDW.

Figure 18 below shows the forest plot representation of studies comparing PDW in sickle cell patients during VOEs and those in the stable phase:

Of the latter, two studies compared PDW in sickle cell patients in VOEs and those in the stationary phase, including a total of 287 participants: 190 patients in VOEs and 97 patients in the stationary phase. The SMD reported using the random‐effects model is −0.09 (−0.35; 0.17), reflecting a slight negative influence of VOEs on PDW. The study reporting the greatest effect is that of Antwi‐Boasiako et al. [10] (−0.38 [−0.99; 0.24]). Heterogeneity between the included studies is *I* ^2^ = 4.2*%*.

##### 3.4.3.4. PCT.

**Figure 19:**
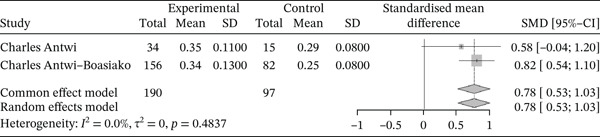
Forest plot showing platecrit.

Figure 19 below shows the forest plot representation of studies comparing PCT.

Of the latter, two studies compared PCT in sickle cell patients during VOEs and those in the stationary phase, for a total of 287 participants, including 190 patients in VOEs and 97 patients in the stationary phase. The SMD reported with the random‐effects model is 0.78 (0.53; 1.03), reflecting a significantly positive influence of VOEs on PCT. The study reporting the greatest effect is that of Antwi‐Boasiako et al. [10] (0.82 [0.54; 1.10]). Heterogeneity between the included studies is *I* ^2^ = 0.0*%*.

## 4. Discussion

SCD is a genetic disorder characterized by inherent clinical manifestations, which are markedly exacerbated by VOEs, thereby affecting hematological parameters. In contrast, the steady‐state phase is defined by the absence of VOEs, with milder clinical symptoms and generally less disrupted biological and hematological parameters [11, 31]. However, interpreting these steady‐state parameters using conventional reference values from learned societies often categorizes them as “abnormal,” despite patients exhibiting clinical stability. This systematic review and meta‐analysis, covering studies from 2000 to 2025, aimed at synthesizing existing data on hemogram profiles in SCD patients. The fundamental clinical utility of this meta‐analysis lies in identifying objective biological signatures of VOEs. Currently, pain management relies on patient self‐assessment, which can be challenging in emergency settings [32]. Our findings are aimed at demonstrating that consistent changes in CBC parameters—specifically significant increases or decreases in certain values in patients during a crisis compared with their steady‐state phase—could serve as a “biological validation” of an acute episode. Standardizing these markers would not only help confirm VOE but also allow monitoring of the resolution kinetics to optimize the timing of hospital discharge.

Analysis of the RBC lineage revealed that VOEs have a significant impact on its parameters. Specifically, our meta‐analysis demonstrated anemia and erythrocytopenia, with SMDs of −0.97 (−1.45; 0.50) and −1.11 (−1.83; −0.38), respectively, reflecting a substantial negative effect on hemoglobin levels. Conversely, VOEs were associated with a positive effect on reticulocyte production (SMD = 0.76 [0.46; 1.06]). These findings are consistent with the understanding that VOEs are strongly linked to RBC destruction and hemolysis, which are key contributors to SCD complications [33]. The progression of VOEs typically involves distinct phases: a prodromal phase (Phase 1) characterized by decreased RBC deformability and increased cell density, followed by hemoglobin reduction (Phase 2) [34], and culminating in biochemical and hematological changes, including anemia and reticulocytosis (Phase 3) [34–36]. Splenic sequestration, a major cause of acute anemia in SCD, further contributes to reticulocytosis by trapping RBCs in the spleen prior to atrophy [14, 31, 37]. Numerous studies corroborate these findings, reporting erythrocytopenia and anemia during the critical phase [38, 39]. For example, Panigrahi et al. [27] in India observed significantly lower mean RBC counts (6.7 ± 2.15 T/L vs. 11.4 ± 4.4 T/L, *p* < 0.05) and hemoglobin levels (6.5 ± 2.27 g/dL vs. 11.39 ± 3.6 g/dL, *p* < 0.05) in patients experiencing crises compared with those in the steady‐state. Similar results have been reported by other researchers [10, 28–30, 40–42].

Moreover, the anemias observed are associated with an elevation of RDW, hypochromia, and reduced HbF. The SMDs reported using the random‐effects model were 0.68 (−0.24; 1.59) for RDW, reflecting a significant positive influence of vaso‐occlusive crises on RDW; −0.90 (2.96; 1.15) for MCH, and 0.01 (−1.40; 1.43) for HbF, reflecting a negative influence of VOEs on these parameters compared with controls. Indeed, Phase 2 of crisis development is often associated with an increase in the distribution width of RBCs and hemoglobin concentration [34]. RDW is a parameter that reflects the degree of heterogeneity in erythrocyte volume (anisocytosis) and is traditionally used in laboratory hematology for the differential diagnosis of anemias [43]. Its elevation in SCD indicates profound dysregulation of erythrocyte homeostasis, involving both impaired erythropoiesis and abnormal RBC survival. This dysregulation can result from a variety of underlying metabolic abnormalities, including telomere shortening, oxidative stress, inflammation, poor nutritional status, dyslipidemia, hypertension, erythrocyte fragmentation, and impaired erythropoietin function [44–46]. Hypochromia in SCD is associated with reduced serum ferritin and transferrin saturation levels, suggesting an imbalance between iron intake and the iron required for erythropoiesis. This imbalance leads to decreased hemoglobin levels in mature RBCs and reticulocytes, resulting in increased production of hypochromic erythrocytes and explaining the characteristic hypochromia observed in patients experiencing sickle cell crises [47, 48]. In addition, the observed variations in MCV during VOEs warrant careful interpretation regarding hydroxyurea (HU) therapy. Although HU typically induces macrocytosis by increasing HbF levels, the relatively low baseline MCV in several included studies suggests a low prevalence of HU use in those specific cohorts, likely due to resource‐limited settings or coexisting iron deficiency. However, our meta‐analysis indicates a trend toward increased MCV during VOE, which could be an indirect marker of reticulocytosis and bone marrow stress. Future primary studies should systematically report HU dosage and adherence to better isolate the independent effect of VOE on erythrocyte indices [49].

Furthermore, the impact of HbF remains debatable, raising questions about the absence of a protective effect in sickle cell patients during crises. Although several hypotheses have been proposed, evidence suggests that VOEs may reduce HbF production in these patients, potentially contributing to more severe and painful episodes [50]. Interestingly, patients with elevated HbF levels may still experience severe disease. This phenomenon is attributed to the uneven distribution of HbF among F‐cells, whereas the overall average HbF remains unchanged. In such cases, the number of F‐cells may be insufficient to effectively inhibit HbS polymerization, despite elevated HbF levels [27, 51].

Furthermore, regarding the white blood cell lineage, leukocytosis was observed along with a moderate increase in its subpopulations, with a SMD reported using the random‐effects model of 0.73 (0.23; 1.24), reflecting a significant positive influence of VOEs on white blood cell production. For neutrophils, the SMD was 0.37 (−0.63; 1.38). Indeed, in Phase 3 of VOEs, biochemical and hematological changes include leukocytosis [34, 35]. Leukocytes, particularly neutrophils, play a critical role in the pathogenesis of VOEs. In mouse models of SCD, neutrophils have been identified as key contributors to microvascular obstruction [52]. Leukocytes are also central to the development of complications associated with SCD [53, 54]. Clinical studies have demonstrated that leukocytosis correlates with disease severity and represents a risk factor for major SCD‐related complications, including stroke, acute chest syndrome, and early mortality [11, 53–55]. For example, Panigrahi et al. [27], in India, reported a significant difference in white blood cell counts between sickle cell patients in crisis (15.1 ± 7.9 G/L) and those in the steady‐state phase (13.86 ± 8.82 G/L, *p* < 0.05). Similarly, Antwi‐Boasiako et al. [10], in Ghana, found higher white blood cell counts in patients during crises (14.5 ± 6.1 G/L) compared with those in the steady‐state phase (8.1 ± 2.2 G/L, *p* < 0.05). Comparable observations have also been reported by other researchers [29, 30].

Regarding the platelet lineage, VOEs appear to exert a variable influence on platelet production, with an increase in platelet count (SMD = 0.78 [0.53; 1.03]) accompanied by a decrease in MPV (SMD = −0.40 [−1.14; 0.34]) and PDW (SMD = −0.09 [−0.35; 0.17]). PDW measures the variability in platelet size and is commonly used to differentiate platelet disorders, such as essential thrombocythemia from reactive thrombocytosis. Diallo et al. [18] and several other authors have reported significantly elevated platelet counts during the critical phase, associated with pronounced clinical manifestations in SCD [10, 29, 30]. Thrombocytosis, particularly when associated with increased PCT, may predispose patients to a hypercoagulable state, characterized by platelet activation and subsequent consumption. This process is markedly amplified during VOEs, with the release of E‐selectin, P‐selectin, soluble ICAM‐1, and tissue factor [57–59]. These changes are generally correlated with increases in MPV and PDW, although this differs somewhat from the observations in our findings. MPV is recognized as a marker of platelet activation and has been strongly associated with a higher frequency of VOEs [59–61]. PDW may therefore serve as a useful parameter to assess the severity of vaso‐occlusive crises in sickle cell patients.

The main strength of this study lies in providing concrete, clinically relevant information regarding the impact of VOEs on each component of the blood count, thereby guiding patient management. Importantly, it highlights the need for prospective studies to establish a standardized database of hematological reference values specific to stable sickle cell patients. Limitations include the meta‐analytic nature of the study, which precludes methodological control over the included studies and may introduce selection bias. Additionally, the relatively small number of studies available limits the ability to fully support the proposed hypotheses for certain parameters. Another limitation of this study is that most of the included studies reported unadjusted data, without accounting for potential confounding factors such as age and sex. Nevertheless, the study provides valuable insights that can inform the management and treatment of sickle cell patients.

## 5. Conclusion

SCD is a genetic disorder characterized by inherent clinical manifestations that are markedly exacerbated by VOEs, leading to significant alterations in hematological parameters. This systematic review and meta‐analysis synthesized studies published over the past 25 years to characterize these hematological profiles and explore their potential clinical utility. Our findings confirm the substantial impact of VOEs on all three blood cell lineages—RBCs, white blood cells, and platelets—as well as their associated indices. VOEs are consistently associated with anemia, erythrocytopenia, microcytosis, hypochromia, reduced HbF levels, elevated RDW, and reticulocytosis, alongside leukocytosis and variable platelet responses. Importantly, these recurrent and measurable changes suggest that hematological parameters could serve as objective biomarkers to support the diagnosis and monitoring of VOEs, complementing clinical assessment, particularly in settings where pain evaluation is challenging. In addition, although abnormalities are less pronounced in steady‐state patients, their values often remain outside conventional reference ranges, highlighting a distinct physiological baseline in this population. Consequently, this study underscores the need for prospective investigations to define standardized hematological reference values and validate their use as reliable tools for diagnosing VOEs and monitoring their progression and resolution, ultimately improving clinical decision‐making and patient care.

## Author Contributions

Romaric De Manfouo Tuono, Ingrid Worti Sulem Yong, and Maryline Seuko Njopwouo ensure the selection of the articles, the extraction of the data, interpreted the data, and contributed to the drafting of the manuscript. Josué Louokdom Simo ensures the statistical analysis of the extracted data; Claude Tagny Tayou and Paul Koki supervised the work. Claude Tagny Tayou and Paul Koki reviewed the paper.

## Funding

No funding was received for this manuscript.

## Disclosure

The lead author, Romaric De Manfouo Tuono, affirms that this manuscript is an honest, accurate, and transparent account of the study being reported; that no important aspects of the study have been omitted; and that any discrepancies from the study as planned (and, if relevant, registered) have been explained. All authors read and approved the final version of the manuscript, and Romaric De Manfouo Tuono had full access to all of the data in this study and took complete responsibility for the integrity of the data and the accuracy of the data analysis.

## Conflicts of Interest

The authors declare no conflicts of interest.

## Data Availability

The data that support the findings of this study are available from the corresponding author upon reasonable request.
